# 

**Published:** 1892-09

**Authors:** 


### CONTENTS.

COMMUNICATIONS.

Infection ..... 413
Diagnosis ..... 416
The Use of Gutta Percha as a Root Canal Filling ..... 425
Syncope and Asphyxia ..... 429
Syphilitic Disease of the Nose ..... 433
Crown and Bridge Work ..... 439
Nerve Stretching in Inveterate Trigeminal Neuralgia ..... 449
The World's Congress Auxiliary of the World's Columbian Exposition ..... 450

PROCEEDINGS.

Missouri State Dental Association ..... 454
West Virginia State Dental Society ..... 455
Report on Necrology ..... 455
Practical Use of Bacteria ..... 457

EDITORIAL.

Educational ..... 458
The World's Columbian Exposition ..... 459
The Pan American Medical Congress ..... 460
Biographical ..... 461
Resuscitation after Drowning ..... 462
Tooth Culture ..... 462
The World's Columbian Dental Congress ..... 462
Death of the Inventor of the Hypodermic Syringe ..... 464

